# Mosquito Vitellogenin Genes: Comparative Sequence Analysis, Gene Duplication, and the Role of Rare Synonymous Codon Usage in Regulating Expression

**DOI:** 10.1673/031.007.0101

**Published:** 2007-01-12

**Authors:** Jun Isoe, Henry H. Hagedorn

**Affiliations:** Department of Entomology and Center for Insect Science, University of Arizona, Tucson, AZ. 85721 USA; ^1^ Present address, Department of Biochemistry and Molecular Biophysics, University of Arizona. Tucson, AZ 85721 USA

**Keywords:** nucleotide sequence analysis, gene duplication, amino acid composition, nonsynonymous and synonymous amino acid substitution, autogeny, anautogeny, *Aedes aegypti*, *Ochlerotatus atropalpus*, *Aedes polynesiensis*, *Aedes albopictus*, *Ochlerotatus triseriatus*, *Culex quinquefasciatus*, *Toxorhynchites amboinensis*, *Anopheles albimanus*

## Abstract

Comparative sequence analysis of mosquito vitellogenin (Vg) genes was carried out to gain a better understanding of their evolution. The genomic clones of vitellogenin genes were isolated and sequenced from all three subfamilies of the family Culicidae including Culicinae (*Aedes aegypti*, *Ochlerotatus atropalpus*, *Ae. polynesiensis*, *Ae. albopictus*, *Ochlerotatus triseriatus* and *Culex quinquefasciatus*), Toxorhynchitinae (*Toxorhynchites amboinensis*), and Anophelinae (*Anopheles albimanus*). Genomic clones of vitellogenin genes Vg-B and Vg-C were isolated from *Ae. aegypti* and sequenced. A comparison of Vg-B and Vg-C, with the previously characterized vitellogenin gene, Vg-A1, suggests that Vg-A1 and Vg-B probably arose by a recent gene duplication, and Vg-C apparently diverged from the two other members of the gene family in an earlier gene duplication event. Two vitellogenin genes orthologous to Vg-C were cloned from a *Cx. quinquefasciatus* DNA library, one of which is truncated at the N-terminal end. Single vitellogenin genes, orthologous to Vg-C, were cloned from the *An. albimanus* and *Tx. amboinensis* libraries. Incomplete sequences orthologous to Vg-B and Vg-C were isolated from the *Oc. atropalpus* library. Only partial sequences were isolated from *Ae. polynesiensis*, *Ae. albopictus* and *Oc. triseriatus.* Inferred phylogenetic relationships based on analysis of these sequences suggest that Vg-C was the ancestral gene and that a recent gene duplication gave rise to Vg-A1 and Vg-B after the separation of the genus *Aedes.*

The deduced amino acid composition of mosquito vitellogenin proteins exhibits higher tyrosine and phenylalanine composition than other mosquito proteins except for the hexamerin storage proteins. Analysis of vitellogenin coding sequences showed that a majority of amino acid substitutions were due to conserved and moderately conserved changes suggesting that the vitellogenins are under moderately selective constrains to maintain tertiary structure. The vitellogenin genes of the three anautogenous mosquitoes, that require a blood meal to develop eggs, had very high synonymous codon usage biases similar to highly expressed genes of other organisms. On the other hand, the vitellogenin genes of autogenous mosquitoes, that develop at least one batch of eggs without a blood meal, exhibited low synonymous codon usage bias. An unusual pattern of synonymous codon usage was observed in the first 15 amino acid residues encoding the signal peptide in the vitellogenin genes, where a high number of rarely used synonymous codons are present. It is hypothesized that rare synonymous codons have selectively accumulated in the signal peptide region to down-regulate the rate of translation initiation in the absence of a blood meal. Real-time PCR gene expression experiments showed that all three *Ae. aegypti* vitellogenin genes were highly expressed after a blood meal, and expressed in non-blood-fed females, males, larvae and pupae at trace levels. Sequences were deposited in GenBank (accession numbers: *Ae. aegypti* Vg-B, AY380797, Vg-C, AY373377; *Oc. atropalpus* Vg-B, AY691321, Vg-C, AY691322; *Ae. polynesiensis* Vg-A1, AY691318, Vg-B, AY691319, Vg-C, AY691320; *Ae. albopictus Vg-A1*, AY691316, Vg-C, AY691317; *Oc. triseriatus* Vg-C, AY691323; *Cx. quinquefasciatus* Vg-C1, AY691324, Vg-C2, AY691325; *Tx. amboinensis* Vg-C, AY691326; *An. albimanus* Vg-C, AY691327).

## Introduction

Vitellogenins are precursors of the yolk proteins, which are phosphoglycolipoproteins utilized in oviparous animals to provide nutrition for the developing embryo. Vitellogenins of many animals have multiple vitellogenin genes including the frog, *Xenopus laevis* (Germond et al. 1984), the chicken, *Gallus gallus*, the nematode, *Caenorhabditis elegans* (Spieth et al. 1991), and several insects including *Drosophila melanogaster* (Wahli 1988), and *Aedes aegypti* (Hamblin et al. 1987). The gene duplication events that gave rise to these gene families are not well understood. Duplicated genes could evolve into pseudogenes or into new genes with novel functions as a consequence of relaxation of functional constraints (Shimeld 1999; Krakauer and Nowak 1999). Pseudogenes encoding non-functional vitellogenin have been isolated from *G. gallus* (Silva et al. 1989), *C. elegans* (Spieth et al. 1985), and the shrimp, *Metapenaeus ensis* (Tsang et al. 2003). The entire open-reading frames of vitellogenin gene sequences in insects have been documented in Dictyoptera (*Blatella germanica*, Martin et al. 1998; *Leucophaea maderae*, Tufail and Takeda 2002; *Periplaneta americana*, Tufail et al. 2000), Hemiptera (*Plautia stali*, *Lee* et al. 2000; *Riptortus clavatus*, Hirai 1998), Lepidoptera (*Bombyx mori*, Yano et al. 1994; *Lymantria dispar*, Adamczyk et al. 1996), Coleoptera (*Anthonomus grandis*, Trewitt et al. 1992), Hymenoptera (*Apis mellifera*, Piulachs et al. 2003; *Athalia rosae*, Kageyama et al. 1994; *Pimpla nipponica* Nose et al. 1997), and Diptera, *Ae. aegypti* Chen et al. 1994; Romans et al. 1995; *Anopheles gambiae* Holt et al. 2002).

Comparative sequence analyses of vitellogenin genes from taxonomically diverse organisms have suggested that vertebrate and invertebrate vitellogenins share a common ancestry for several reasons. First, the positions of some of the cysteines in the primary structure of vitellogenins are highly conserved, especially at the C-terminal region (Trewitt et al. 1992; Hagedorn et al. 1998; Sappington and Raikhel 1998). The conserved positions of cysteines are likely involved in the formation of a complex tertiary structure of vitellogenins. The tertiary structure of Lamprey vitellogenin has been determined by X-ray crystallography (Raag et al. 1988; Thompson and Banaszak 2002). Second, the position of some introns in vertebrate and invertebrate vitellogenin nucleotide sequences are well conserved (Nardelli et al. 1987; Trewitt et al. 1992; Hagedorn et al. 1998). Third, although sequence analysis has revealed extensive sequence divergence between vitellogenins of all organisms, the existence of some limited conserved regions suggest that they are related (Wahli 1988; Byrne 1989, Hagedorn et al. 1998; Sappington and Raikhel 1998). However, many amino acid substitutions were found to be conserved changes in the physical and chemical properties of the amino acids, implying that the overall tertiary structure of vertebrate and invertebrate vitellogenin genes are highly constrained and conserved (Trewitt et al. 1992; Hagedorn et al. 1998). Fourth, polyserine regions are present in many vertebrate and invertebrate organisms (Romans et al. 1995; Hagedorn et al. 1998; Sappington and Raikhel 1998). Taken together, this evidence suggests that vertebrate and invertebrate vitellogenins share a common ancestry.

The regulation of expression of highly expressed proteins sometimes involves the use of specific synonymous codons. When numerous gene sequences in *E. coli* and *Saccharomyces cerevisiae* were analyzed, a pronounced bias in patterns of synonymous codon usage was observed, especially in highly expressed genes where many amino acid residues are selectively coded by a single preferential synonymous codon (Kurland 1991; Ikemura 1985). The corresponding isoaccepting tRNAs of preferentially used synonymous codons in highly expressed genes were also found to be very abundant compared to rarely used synonymous codons. The abundance of each isoaccepting tRNA differs significantly in *E. coli* (Gouy and Gautier 1982; Ikemura 1985), and *D. melanogaster* (Moriyama and Powell 1997; Powell and Moriyama 1997). The abundance of tRNA isoaccepting species can be different in different tissues in eukaryotes and can be regulated differentially in response to developmental and physiological processes in bacteria (Emilsson and Kurland 1990), in plants (Sreenagesh and Jayabaskaran 1996), in arthropods (Chevallier and Garel 1979; Candelas et al 1990), and in humans (Kanduc et al. 1997). Thus, as a general rule in both prokaryotes and eukaryotes, highly expressed proteins tend to be selected for preferential usage of optimal synonymous codons for translational efficiency and/or accuracy.

In this study, the entire coding region of the vitellogenin genes from three anautogenous mosquitoes, *Ae. aegypti*, *Culex quinquefasciatus* and *Anopheles albimanus*, and two autogenous mosquitoes, *Ochlerotatus atropalpus* and *Toxyrhynchites amboinensis*, and several partial vitellogenin coding sequences from three other anautogenous mosquitoes (*Aedes polynesiensis*, *Aedes albopictus*, and *Ochlerotatus triseriatus*) were cloned and sequenced to gain a better understanding of 1) vitellogenin gene duplication events within the mosquito lineage, 2) amino acid composition, and 3) the degree of nonsynonymous and synonymous amino acid substitutions.

**Table 1.**
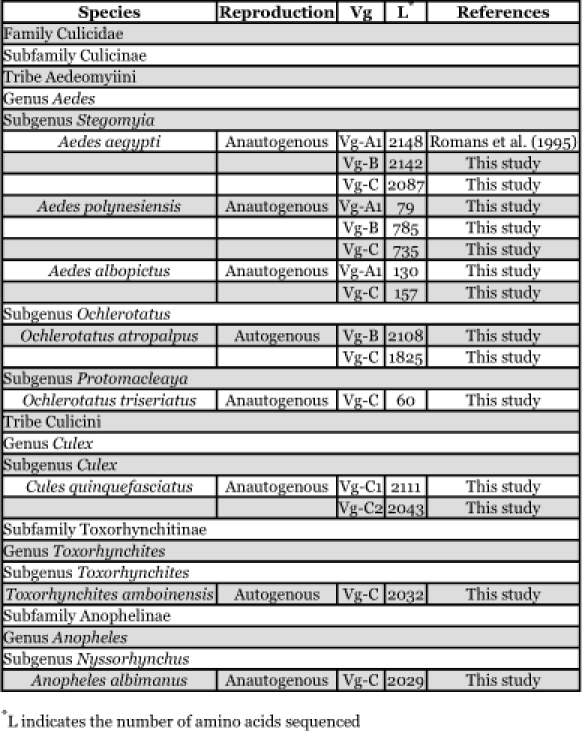
Classification and reproductive strategies of mosquitoes

## Materials and Methods

Mosquitoes used in this study were selected based on taxonomic relationships representing all three mosquito subfamilies with different reproductive strategies (i.e. autogenous and anautogenous reproduction). The taxonomic classification and reproductive strategies of the mosquitoes studied are shown in Table 1. Reinhart et al. (2004) have described a revision of the tribe Aedini and renamed some members of the genus *Aedes*; *Ae. aegypti* to *Stegomyia aegypti*, and *Ae. albopictus* to *St. albopictus.* This change has been controversial (Savage 2005) (see also http://wrbu.si.edu/forums/viewtopic.php?t=9). We have retained the earlier classification.

### Genomic DNA library construction

Genomic DNA was isolated from one anautogenous mosquito species, *Cx. quinquefasciatus*, kindly provided by J. Chaney at the University of California, Riverside and two autogenous mosquito species, *Oc. atropalpus* and *Tx. amboinensis* that were kindly provided by D. Wheeler at the University of Arizona, Tucson, Arizona and F. Mahmood at Rutgers University, New Brunswick, New Jersey, respectively. Fresh or alcohol preserved mosquitoes were used to isolate genomic DNA (Sambrook et al. 1989). The high molecular weight genomic DNA was subjected to partial digestion with *Sau*3AI. The partially digested genomic DNA was separated either on a spharose gradient or low melting point agarose gels, from which bands between 6.0 and 10.0 kb were excised and purified using GELase (Epicentre Technologies, http://www.epicentre.com/main.asp). The purified genomic DNA was ligated using a *Bam*HI predigested λ ZapExpress vector and packaged using Gigapack III Gold Packaging Extract from Stratagene (http://www.stratagene.com/). All three unamplified libraries had ∼5 × 106 plaque forming units. Genomic DNA libraries from *Ae. aegypti* constructed in Lambda Dash® II vector, from *An. albimanus* constructed in Lambda EMBL 3 vector, and from *Ae. polynesiensis*, *Ae. albopictus*, and *Oc. triseriatus* constructed in a λ ZapExpress vector were provided by A. A. James (University of California, Irvine), M. Kidwell (University of Arizona, Tucson, Arizona), and R. Nussenzveig (University of Arizona, Tucson, Arizona), respectively.

### Screening genomic DNA libraries

The *Ae. aegypti* genomic DNA library was probed using DNA probes containing conserved vitellogenin coding sequences (Hagedorn et al. 1998): Vg-A1 nucleotides 2425–4338, GenBank accession # L41842 (Romans et al. 1995), labeled with digoxigenin-11-dUTP using the random priming method (Roche Molecular Biochemicals, www.roche.com). Approximately 50,000–300,000 plaques were screened. *Ae. aegypti* Vg-B and Vg-C DNA probes were chosen to contain the conserved region of vitellogenin genes described above that were then used to probe the *Oc. atropalpus*, *Cx. quinquefaciatus*, *Tx. amboinensis*, and *An. albimanus* DNA libraries. Approximately 50,000–300,000 plaques were screened. Approximately 50,000–200,000 plaques were screened for the *Ae. polynesiensis*, *Ae. albopictus*, and *Oc. triseriatus* libraries. Genomic DNA libraries were constructed in a Lambda Dash® II vector (Stratagene, www.stratagene.com) kindly provided by A. A. James (University of California, Irvine). The plaques were transferred to nylon membranes (Micron Separations Inc., www.stratagene.com). The membranes were denatured, neutralized and DNA cross-linked using a Strata-linker (Strategene). Hybridization was performed overnight at 550C using a Gene Roller (Savant Instruments Inc, www.combichemlab.com). Positive plaques were detected colormetrically using anti-digoxygenin conjugated with alkaline phosphatase, with nitroblue tetrazolium and 5-bromo-4-chloro-3-indolyl phosphate as substrates. All positive plaques were further screened to purify each positive plaque. Phage DNAs for each putative vitellogenin gene cloned were extracted and purified using methods described by Sambrook et al. (1989). Phages were precipitated with PEG 8000 and NaCl overnight instead of for one hour. Phage DNAs were subsequently digested with restriction enzymes and electrophoresed in 1.0% agarose gels in TAE buffer. DNA fragments encoding vitellogenin genes were subcloned into the pBluescript SK+plasmid vector (Strategene), and plasmid DNAs were isolated by plasmid DNA Miniprep (Promega, www.promega.com/). Transformation was carried out using DH5α competent cells (Life Technologies Inc., www.lifetech.com). All sequencing was performed at the University of Arizona Sequencing Facility using an automatic sequencer (Model 373, Applied Biosystems, appliedbiosystems.com). Sequences were determined from both strands. Sequences were deposited in GenBank (accession numbers: *Ae. aegypti* Vg-B, AY380797, Vg-C, AY373377; *Oc. atropalpus* Vg-B, AY691321, Vg-C, AY691322; *Ae. polynesiensis* Vg-A1, AY691318, Vg-B, AY691319, Vg-C, AY691320; *Ae. albopictius Vg*-*A1,* AY691316, Vg-C, AY691317; *Oc. triseriatus* Vg-C, AY691323; *Cx. quinquefasciatus* Vg-C1, AY691324, Vg-C2, AY691325; *Tx. amboinensis* Vg-C, AY691326; *An. albimanus* Vg-C, AY691327).

### Determination of intron and 3′ untranslated region sequences

Positions of introns for each vitellogenin gene were determined by comparison of genomic and cDNA sequences. Fat body preparations (which include the abdominal epidermis, heart, nerve cord and associated tissues) were dissected from female mosquitoes 24 hours after a blood meal and were frozen in liquid nitrogen. Total RNA was isolated using the guanidine thiocyanate method and an RNaid Kit (BIO 101, www.qbiogene.com) and stored at -800C until use. The potential genomic DNA contamination in total RNA preparations was degraded with DNaseI prior to reverse transcription, followed by enzyme denaturation at 6500. 1 µg of the total RNA was subjected to reverse transcription using a universal primer for the three *Ae. aegypti* vitellogenin genes at 420C (Table 2) using Superscript reverse transcriptase (Life Technologies, www.invitrogen.com). The RNA template was subsequently removed by RNase H (Life Technologies). Polymerase chain reaction (PCR) was performed using gene-specific primers that were designed to flank each potential intron (Table 2). The PCR products were separated on agarose gels and the expected bands were purified using a Sephaglas BandPrep Kit (Pharmacia, www.Pharmacia.com). The PCR products were inserted into TA cloning vectors (Invitrogen, www.invitrogen.com/) following the manufacture's instructions, and positive clones were sequenced.

**Table 2.**
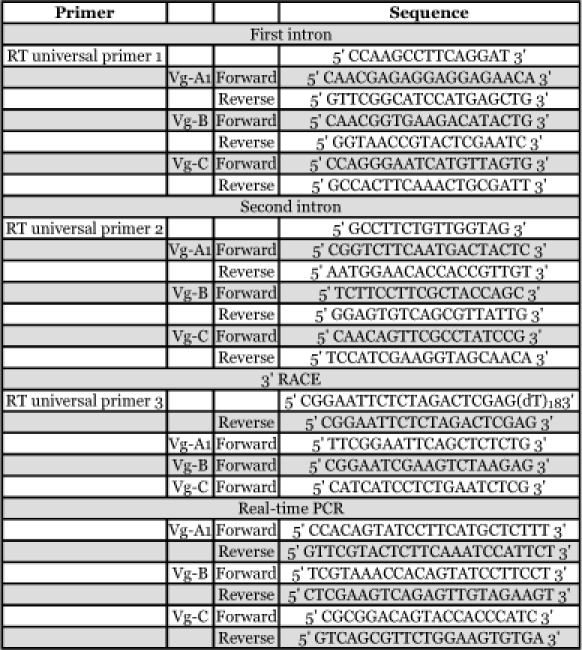
Oligonucleotide primer sequences used for analysis of *Ae. aegypti* vitellogenin genes.

Rapid amplification of cDNA ends (3′ RACE) was carried out to determine the 3′ untranslated region for each vitellogenin gene. Total RNA was isolated from female fat body 24 hours after a blood meal as described above. The first strand cDNA was synthesized from the total RNAs using an Oligo-(dT) containing an adapter primer (Table 2). PCR was performed on cDNA using gene-specific primers and an adapter primer. The PCR products were analyzed and cloned as above.

### Gene expression pattern in *Ae. aegypti* by real-time PCR

Real-time RT-PCR was performed to quantify differences in vitellogenin gene expression in *Ae. aegypti* after a blood meal. Fat body preparations, midgut, and ovary tissues were dissected from unfed and fed mosquitoes at specific times after a blood meal. Isolated tissues were stored in RNAlater (Ambion, www.ambion.com) at -80°C until use. Total RNA was extracted TRIzol reagent (Invitrogen) according to the manufacture's instructions. Total RNA was also isolated from whole larvae, male and female pupae, and male adult. Prior to synthesizing cDNA from each sample, 1 µg total RNA was first treated with RNase-free DNaseI to ensure complete degradation of potential genomic DNA contamination, followed by the addition of 25 mM EDTA and heat denaturation of the enzyme. Reverse transcription was carried out using oligo-(dT)20 primer and reverse transcriptase (NEB, www.neb.com) in a 20 µl reaction volume at 37°C for 1 hour followed by termination of the reaction at 70°C for 20 min. The final volume of cDNA was increased to 200 µl. To minimize potential variation in reverse transcriptase efficiency, all cDNA syntheses were carried out simultaneously. To design optimized gene-specific sense and antisense oligonucleotide primers without primer-dimer formation and self-priming formation, we used OLIGO software (version 6, Molecular Biology Insights, www.oligo.net/) for each vitellogenin gene (Table 2). Nucleotide pairwise comparison was performed using the GAP program in the GCG (Genetic Computer Group, www.accelrys.com/products/gcg/) to ensure that the primers anneal exclusively to the DNA of specific vitellogenin genes. Oligonucleotide primers were custom made by OPERON (http://www.operon.com/)

The real-time RT-PCR was carried out in the ABI PRISM 7700 Sequence Detection System (Applied Biosystems) according to the manufactures instructions in a 96-well microtiter plate with a 10 µl reaction volume containing 5 µl SYBR Green PCR Master Mix (Applied Biosystems), 3 µl of each primer set (0.5 µM final concentration), and 2 µl of cDNA templates. Each sample was run in triplicate. Negative controls without template were performed in each run. PCR conditions: preincubation was performed for 10 min at 95°C to denature the target DNA and activate AmpliTaq Gold DNA Polymerase; DNA was amplified for 40 cycles of 15 sec at 95°C and 1 min at 60°C. Data were analyzed by ABI software, Version 1 (www.appliedbiosystems.com/).

### Sequence analysis

Multiple alignment of protein sequences from translation of nucleotide sequences was performed using PILEUP of GCG Wisconsin Software Package (version 10.0-UNIX) and subsequently improved by eye using MacClade (test version 4.0a11, Maddison and Maddison 1999). Pairwise comparisons was performed to estimate sequence divergence of *Ae. aegypti* vitellogenins using the PAUPµ 4.0b2 package (Phylogenetic Analysis Using Parsimony, Swofford 2000). The extent of amino acid substitutions were categorized based on physicochemical properties of amino acids (Grantham 1974).

Additional gene sequences analyzed in this study were obtained from the GenBank database. Putative ribosomal protein genes were retrieved from the *Anopheles gambiae* genome by BLAST searches. The effective number of codons, ENC, was used to measure overall synonymous codon usage bias (Wright 1990). Values of ENC can range between 20, in an extremely biased gene, where only one codon is used for each amino acid, and 61, where all synonymous codons are used with the same probability. ENC, GC content, and GC3 (G+C content at the 3rd position of the synonymous codon) were calculated for each gene using CodonW (Peden 1997). A χ2 test of homogeneity of base frequency across taxa was performed using the PAUP* 4.0b2 package (Swofford 2000) to determine the level of bias at each codon position.

## Results

### Cloning of vitellogenin genes

The screening of the *Ae. aegypti* genomic DNA library was conducted at low stringency to clone different members of the vitellogenin (Vg) gene family using an *Ae. aegypti* Vg-A1 DNA probe corresponding to nucleotides 2425–4338 (accession # L41842, Romans et al. 1995) that contains the most conserved domains within the insect vitellogenin genes (Hagedorn et al. 1998). Four genes were previously isolated from an *Ae. aegypti* genomic DNA library and restriction mapped (Gemmill et al. 1986; Hamblin et al. 1987). Based on the comparisons of the restriction sites present in the four vitellogenin genes, Vg-A1, A2, B, and C, isolated in this study, and the previously mapped clones, the same four vitellogenin genes had been cloned.

**Figure 1.**
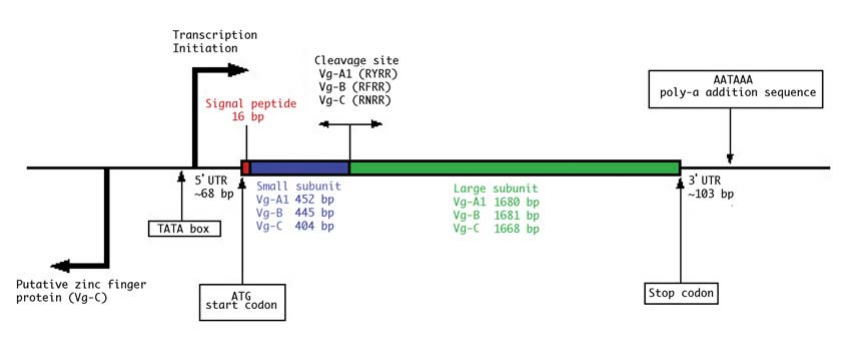
Generalized genomic organization of *Aedes aegypti* vitellogenin genes. The number in parenthesis indicates the number of amino acid residues.

Genomic libraries of *Ae. albopictus*, *Ae. polynesiensis*, *Oc. atropalpus*, *Oc. triseriatus*, *Cx. quinquefasciatus*, *Tx. amboinensis* and *An. albimanus* were subsequently screened at low stringency using *Ae. aegypti* Vg-B and Vg-C DNA probes, which were chosen to contain the conserved region of vitellogenin genes. Vitellogenin genes cloned from each species were also used to rescreen the library to clone possible additional members of the vitellogenin gene families. The vitellogenin genes sequenced from these mosquito species were classified as orthologous to one of the members of vitellogenin gene family in *Ae. aegypti* as judged by sequence identity, molecular signatures such as insertion and deletion events, and phylogenetic analysis of vitellogenin gene sequences.

### Structure and sequence of the *Ae. aegypti *vitellogenin genes

DNA sequences in part of the coding and upstream regulatory regions of Vg-A1 and Vg-A2 of *Ae. aegypti* had high identity including the coding region (98.5% nucleotide identity with synonymous substitutions) and the upstream region (97.5%), suggesting that these two genes are allelic variants or derived from a recent gene duplication. Further analysis of Vg-A2 was not pursued.

**Figure 2.**
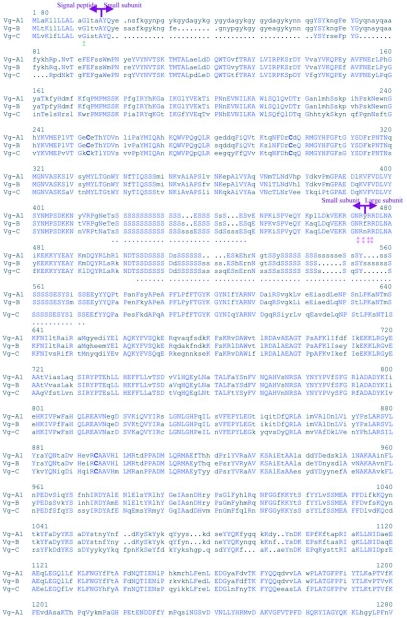
A multiple deduced amino acid sequence alignment of three members of vitellogenin family in *Aedes aegypti.* The conserved amino acid residues are capital letters. The conserved cysteine residues are marked with bold letters. An intron splice site is marked by Ï. Polyserine regions are underlined by dots (…). The symbol (ˆ) indicates a region where no nonsynonymous substitutions occur between Vg-A1 and Vg-B. The first set of arrows indicate the end of the signal peptide and the beginning of the small subunit. The second set of arrows indicate the position of the cleavage site where the ‘small’ subunit ends and the ‘large’ subunit begins.‡‡‡‡ indicate the cleavage sequence (RXRR) between the large and small subunits.

Figure 1 shows the generalized structural organization of the mosquito vitellogenin genes. The intron and exon structures of Vg-B and Vg-C of *Ae. aegypti* were predicted based on their consensus 5′ and 3′ splice sites and by comparison to Vg-A1 cDNA (Chen et al. 1994). Total RNA isolated from fat body preparations of blood fed females was subjected to RT-PCR using gene-specific primers for Vg-B and Vg-C. The sequences derived from each PCR product established precise intron splicing sites for Vg-B and Vg-C, exactly as had been found previously for Vg-A1 (Romans et al. 1995). The 3′ untranslated region of Vg-B and Vg-C was determined by 3′ RACE. As described for Vg-A1, Vg-B and Vg-C had short 3′ untraslated regions (110 and 84 bp, respectively), having no sequence similarity to one another or to Vg-A1. The transcription initiation site and 5′ untranslated regions for Vg-B and Vg-C were predicted based on the sequence comparison with Vg-A′ (Figure 2).

**Table 3.**
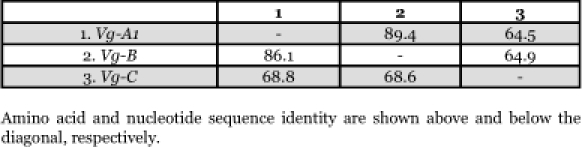
Uncorrected sequence identity (%) of three members of the vitellogenin gene family of *Aedes aegypti*

**Continued:**
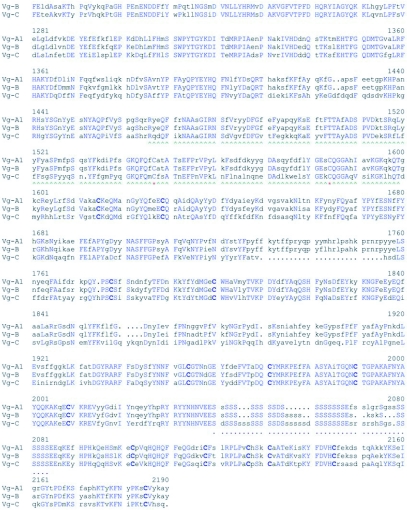

The deduced amino acid sequence alignment of Vg-A1, Vg-B and Vg-C of *Ae. aegypti*, is shown in Figure 2, and the unconnected pairwise sequence divergence of amino acid and nucleotide sequences is presented in Table 3. Sequence comparison of the coding regions of Vg-A1 and Vg-B showed high identity (86.1% and 89.4%) for the nucleotides and deduced amino acids, respectively. Romans et al. (1995) showed that the genomic sequence of Vg-A1 and the cDNA sequence of Chen et al. (1994) showed no significant differences except that the Vg-A1 sequence has an extra imperfect repeat of amino acid sequences near the N-terminal region of the large subunit. Vg-B has two imperfect repeats of this sequence. Sequence comparisons between Vg-C and other members of the vitellogenin gene family showed that Vg-C is more different from Vg-A1 and B, and that nucleotide identity was higher than amino acid identity, suggesting the presence of more amino acid substitutions (Table 3).

### The vitellogenin genes of eight other mosquito species

The vitellogenin genes of eight other mosquitoes were also examined. Table 1 provides information about the relationships between these mosquitoes. Appendix 1 shows the deduced amino acid sequence alignment of these genes. Two incomplete sequences coding for vitellogenin genes orthologous to *Ae. aegypti* Vg-B and Vg-C were cloned from the autogenous *Oc. atropalpus* mosquito. Screening the *Cx. quinquefasciatus* library resulted in the cloning of two different members of the vitellogenin gene family that appear to be orthologous to Vg-C of *Ae. aegypti* and were designated as Vg-C1 and Vg-C2. The Vg-C1 clone contains a complete coding sequence of the gene, whereas the Vg-C2 clone has all of the molecular signatures of a vitellogenin gene but no frame shifts and stop codons are present. However, this gene is truncated in such a way that 39 amino acid residues at the N-terminal are missing from the coding region. Partial sequences coding for the N-terminal region of vitellogenin genes from three anautogenous mosquitoes, *Ae. polynesiensis* (orthologous to *Ae. aegypti* Vg-A1, Vg-B, and Vg-C), *Ae. albopictus* (orthologous to *Ae. aegypti* Vg-A1 and Vg-C) *and Oc. triseriatus* (orthologous to *Ae aegypti* Vg-C) were also sequenced (Appendix 1).

Intensive screening of *An. albimanus* and *Tx*. *amboinensis* libraries resulted in the isolation of a single vitellogenin gene for each of these species. Subsequent screening of both libraries with homologous probes did not yield additional members of vitellogenin genes of either species. The vitellogenin genes sequenced from *An. albimanus* and *Tx. amboinensis* appear to be orthologous to *Ae. aegypti* Vg-C (Appendix 1). For example, no duplicated imperfect repeats were found between Vg-C of *Ae. aegypti* and the vitellogenin genes from *An. albimanus* and *Tx.* *amboinensis.* Also the alignments show that there are several stretches of amino acid deletions and insertions in identical positions in Vg-C of *Ae. aegypti* and the vitellogenin genes in *An. albimanus* and *Tx. amboinensis* that are not present in Vg-A1 and Vg-B of *Ae. aegypti*, suggesting that the *Ae. aegypti* Vg-C and the vitellogenin gene cloned from these two species probably shared a recent common ancestor, and that Vg-A1 and Vg-B of *Ae. aegypti* are distantly related to them. Comparison of the incomplete pairwise sequence divergence of Vg-C genes orthologous to Vg-C of *Ae. aegypti* shows that they have relatively high homology with Vg-C of *Ae. aegypti* (Table 4).

**Table 4.**
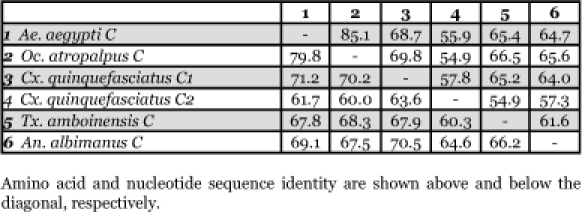
Uncorrected sequence identity (%) of six mosquito vitellogenin Vg-C genes.

### Amino acid composition of vitellogenin proteins

There are no dramatic differences in the amino acid composition between the mosquito vitellogenins (Table 5). Three polyserine regions are present (Appendix 1). Most are rich in tyrosine (Y) and phenylalanine (F), however, Vg-C1 and Vg-C2 of *Cx. quinquefasciatus* have a lower number of these two amino acids compared to the other mosquitoes (Table 5). The reduction is more prominent in the truncated gene, Vg-C2, than in Vg-C1. A reduction in the number of serine residues in *An. albimanus* Vg-C by about 30% is also apparent. The deduced amino acid composition of vitellogenin proteins from other insects is also shown in Table 5. Interestingly, none of the vitellogenin proteins of other insects have a high content of tyrosine and phenylalanine.

**Figure 3.**
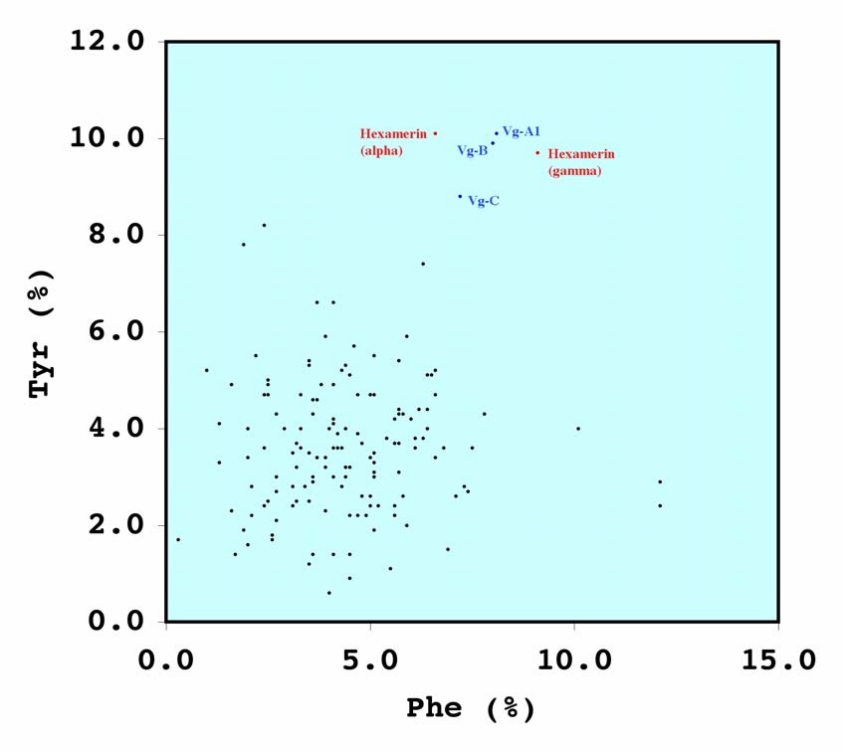
Distribution of the compositional percentage of phenylalanine and tyrosine aromatic amino acid residues of *Aedes aegypti* proteins. The location of the vitellogenins and hexamerin proteins of *Ae. aegypti* are shown (Gordadze et al. 1999).

*Ae. aegypti* vitellogenin proteins show a biased amino acid composition compared to 144 other *Ae. aegypti* proteins available from the GenBank database (Table 6). In general, the aromatic amino acids tyrosine and phenylalanine that are rich in most mosquito vitellogenin proteins contribute a relatively low proportion of amino acids in all other proteins examined, with the exception of the hexamerin proteins, which are also rich in methionine (Figure 3). The hexamerins of the mosquitoes, *Oc. atropalpus* (AAL29455), *An. gambiae* (U51225), and larval *Ae. aegypti* (Gordadze et al. 1999) are also high in tyrosine and phenylalanine.

**Table 5.**
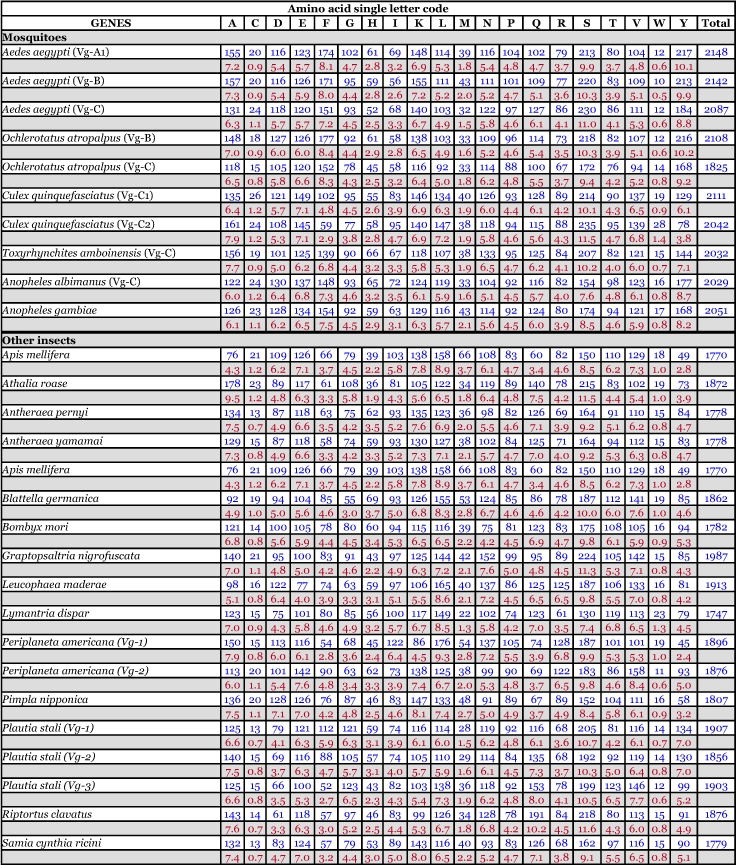
Amino acid composition of insect vitellogenin proteins, not including mitochondrial proteins. Blue = # of amino acids. Red = percent of total # of amino acids.

### Conservative and non-conservative amino acid substitutions in *Ae. aegypti* Vg

Grantham (1974) scored differences between amino acid substitutions based on the physical and chemical properties of amino acids. He categorized amino acid changes as conserved (score < 50), moderately conserved (50 < score < 100), moderately radical (100 < score < 150), and radical substitution (score > 150). Using this scoring scheme, the entire coding regions of vitellogenin genes were divided into 30 amino acid blocks each based on the alignment of the three *Ae. aegypti* vitellogenin proteins (Figure 2) and compared to assess the degree of amino acid substitutions.

**Table 6.**
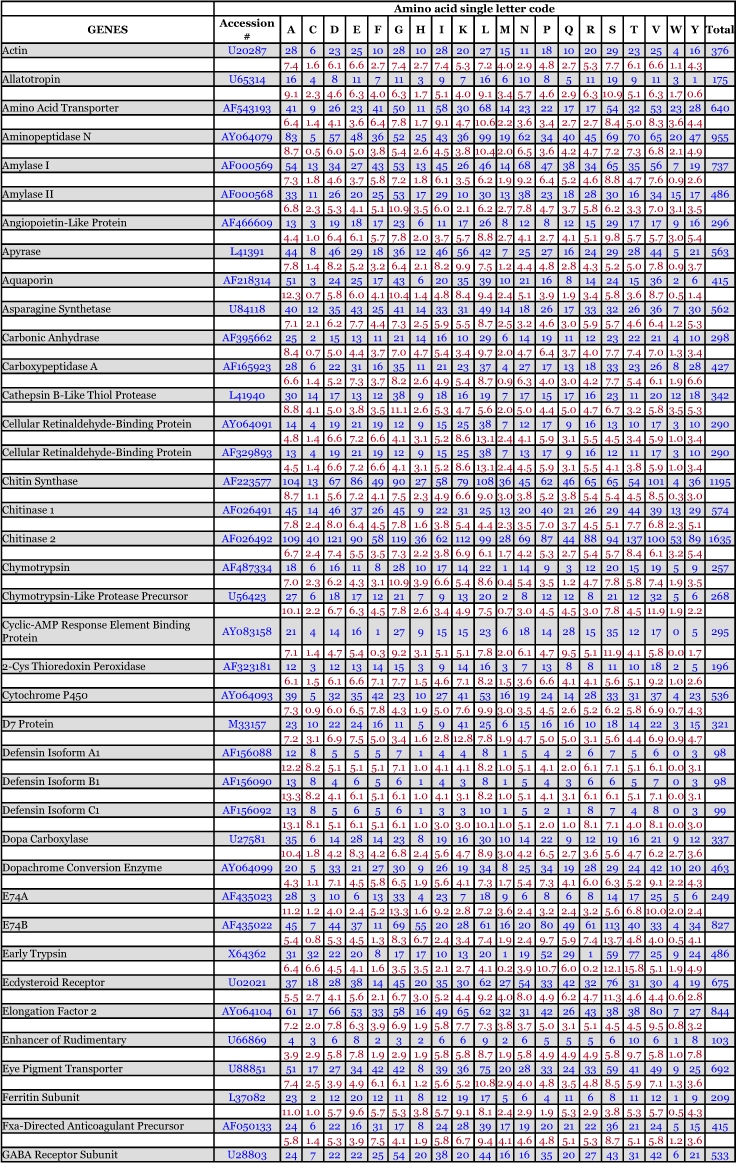
Amino acid composition of *Aedes aegypti* proteins, not including mitochondrial proteins. Blue = # of amino acids. Red = percent of total # of amino acids.

**Continued:**
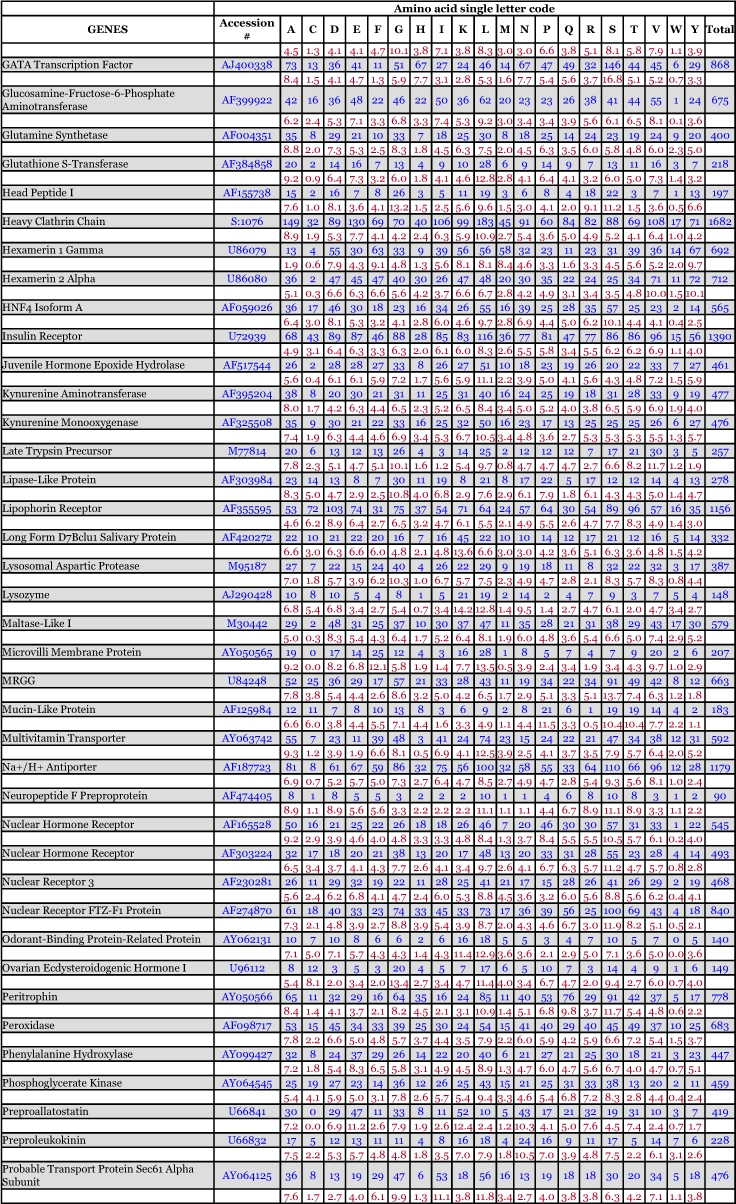

**Continued:**
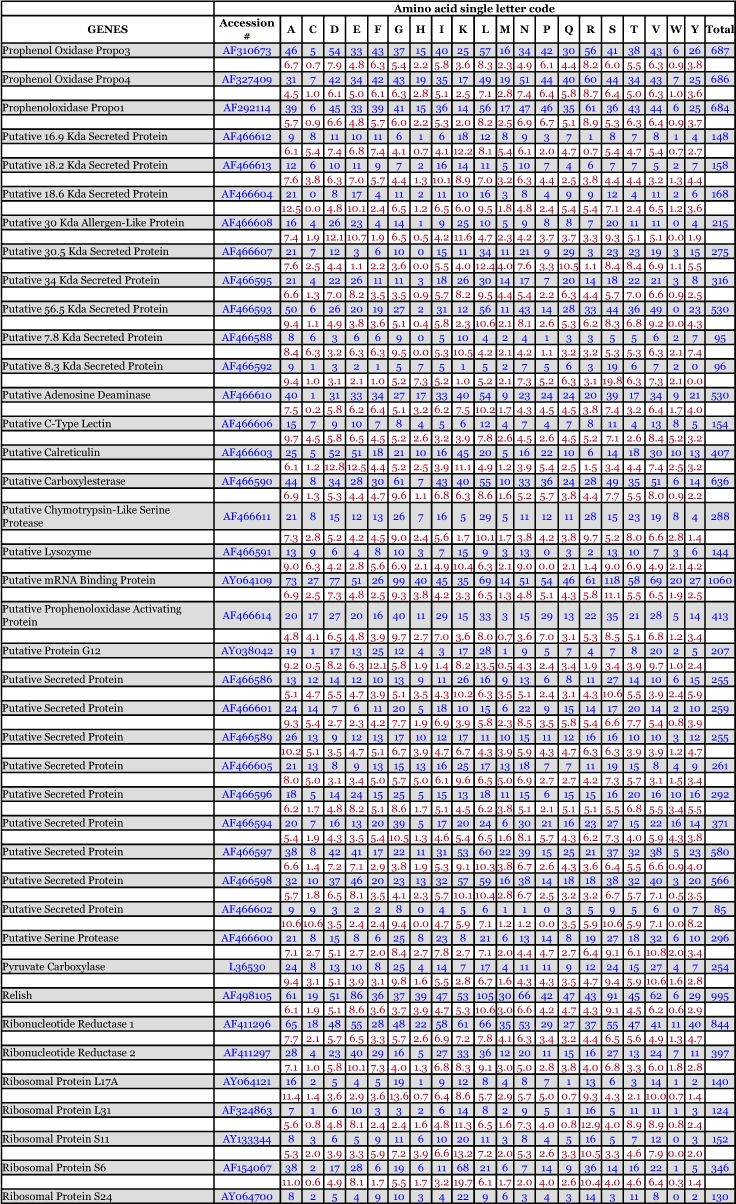

**Continued:**
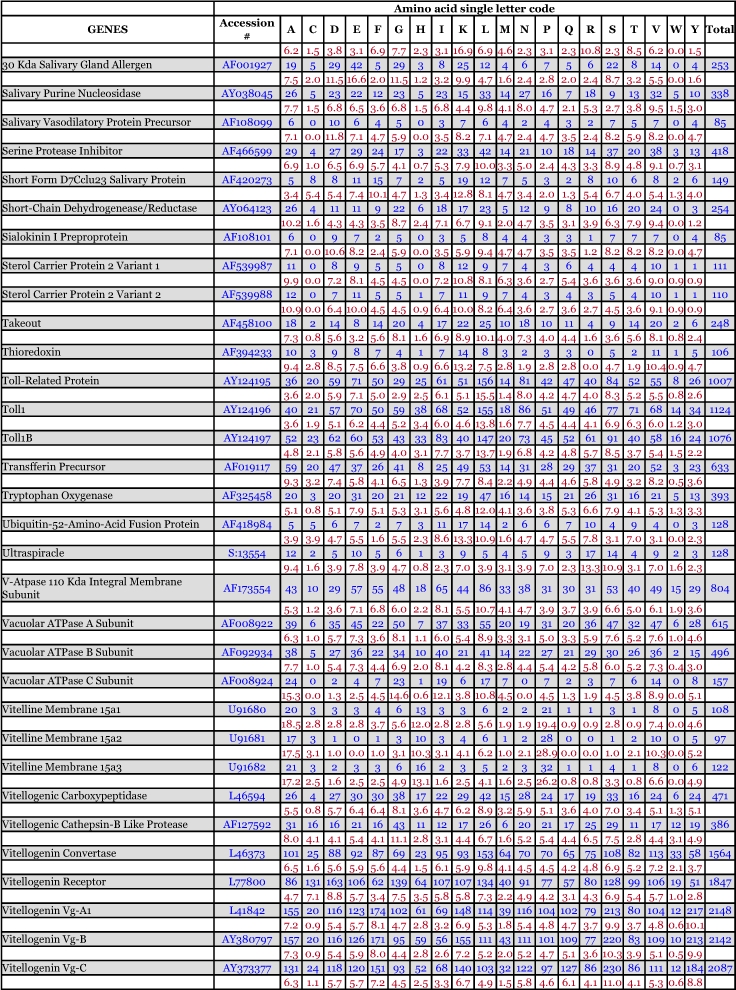

**Figure 4 (Part 1).**
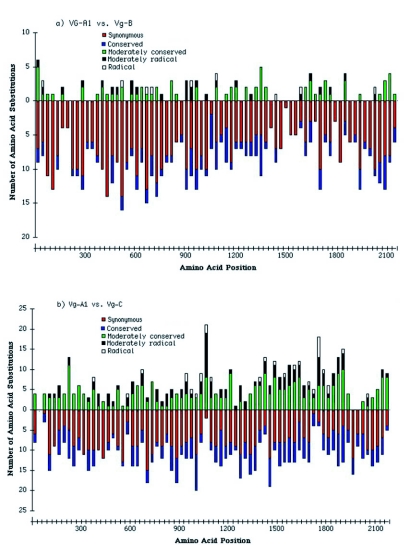
Degree of nonsynonymous substitutions in pairwise comparison of the *Aedes* *aegypti* vitellogenin genes; (a) Vg-A1 and Vg-B, (b) Vg-A1 and Vg-B, and (c) Vg-B and Vg-C. The meaning of the bars is indicated above the figure.

A substitutional comparison of Vg-A1 and Vg-B of *Ae. aegypti* showed that among 227 amino acid changes, the majority (84.1%) resulted in conserved/moderately conserved substitutions (Table 7). Moderately radical and radical amino acid substitutions (15.9%) were distributed throughout the sequences. Nonsynonymous amino acid substitutions were absent from a 136 amino acid sequence of Vg-A1 and Vg-B (residues 1466∼1599 in Figure 2) suggesting the action of selective constraints in this region. A substitutional comparison of Vg-A1 and Vg-B to Vg-C also showed that most amino acid changes were due to conserved and moderately conserved changes in amino acid properties (Table 7). However, the number of amino acid substitutions increased dramatically from 226 amino acid changes in Vg-A1 and Vg-B (Figure 4a) to an average of 725 changes in Vg-C, indicating that Vg-C is significantly different from Vg-A1 and Vg-B (Figure 4b, c). Vg-C showed radical and moderately radical amino acid substitutions distributed throughout the protein, except in the least conserved region (1041∼1101) where a large number of these amino acid changes were present, suggesting that this region is less constrained (Figure 4b, c).

The extent of nonsynonymous substitutions was also examined between the Vg-C orthologs of different mosquito species. As shown in Table 7, most nonsynonymous substitutions resulted from conserved and moderately conserved substitutions (average of 43.6 and 41.3%, respectively). Only an average of 2.7% of changes were due to radical amino acid substitutions, suggesting that dramatic changes in amino acid properties in most positions could be detrimental to the function of the vitellogenin proteins. An analysis of intragenic positions of nonsynonymous substitutions shows that most radical and moderately radical changes appear to be randomly distributed.

### Synonymous codon usage

**Figure 5.**
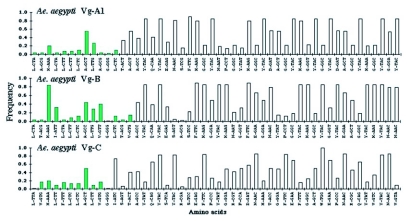
Observed frequency of the first 50 codons in the *Aedes aegypti* vitellogenin genes, excluding the first initiator methionine. Green bars show the codons of the signal peptide, the first 11–14 amino acids, excluding the first methionine. Short bars indicate rare codons.

Synonymous codon usage bias was examined in vitellogenin genes from insects and other organisms (Table 8) by determining the effective number of codons (ENC). Vitellogenin genes of mosquitoes have a codon bias that ranged from ENC 32.3 (*An. albimanus*) to 50.7 (*Tx. amboinensis*). Other insect vitellogenin and yolk protein genes also have a wide range of codon usage bias, varying from *D. melanogaster* yolk protein 1 (ENC: 32.6) to *S. cynthia ricini* (ENC: 56.4), with varying GC content at the third codon position. Vitellogenin genes from animals other than insects also show varying degrees of codon usage bias. The intragenic position of synonymous codon usage throughout the entire coding region of the vitellogenin genes was examined. A bias for codon usage was observed in the region encoding the signal peptide of *Ae. aegypti* (Figure 5), and all of the other mosquito vitellogenin genes. In this region, which includes 11–15 codons, excluding the first methionine, a high number of rarely used synonymous codons were present. None of the other, non-mosquito, vitellogenin genes shown in Table 8 had a cluster of rare  synonymous codons in the signal peptide region, except for the N-terminal region of *C*. *elegans* vit-5, which has a few rare synonymous codons (Speith et al. 1985).

**(Part 2):**
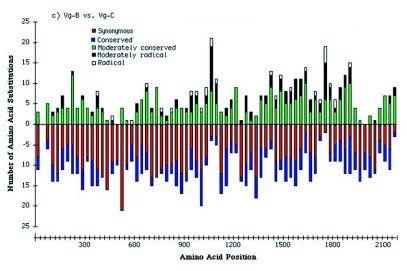

**Figure 6.**
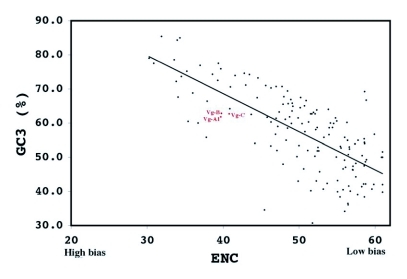
Correlation of synonymous codon usage in 150 *Aedes aegypti* genes. ENC = effective number of codons, GC3 = GC content in the third position. Shown in red are the vitellogenin genes. r^2^ = 0.5235.

**Figure 7.**
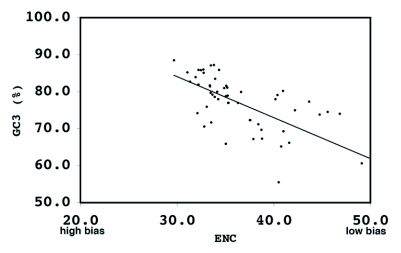
The correlation of synonymous codon usage bias and GC3 *of Anopheles gambiae* ribosomal genes. r^2^ = 0.4606.

By way of comparison, the complete sequences of 150 *Ae. aegypti* genes were analyzed for synonymous codon usage bias (Table 9). The vitellogenin genes showed a pattern of codon usage that is among the most highly biased of all known complete *Ae. aegypti* genes based on ENC. A positive correlation (r^2^ = 0.5235) was found between GC content and the degree of synonymous codon usage bias measured by ENC (Figure 6). Thus, the synonymous codon usage bias of vitellogenins probably reflects the biased GC3. Highly expressed proteins in general have higher selective constraints on synonymous codon choices for translational efficiency and/or accuracy. For example, most of the highly expressed ribosomal genes *of An. gambiae* also show a high synonymous codon usage bias with a biased GC3 (r^2^ = 0.4606) (Table 10 and Figure 7).

**Table 7.**
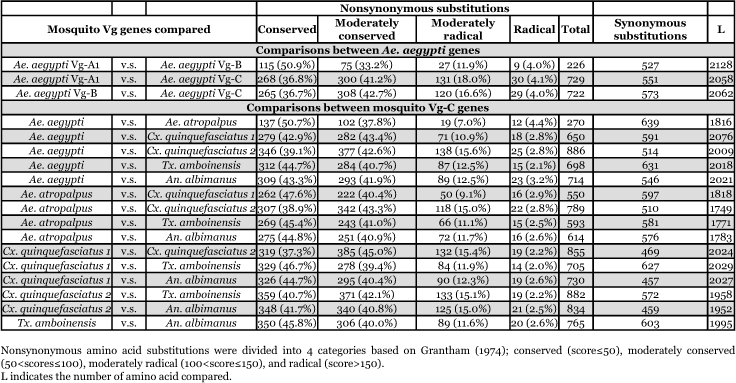
Degree of nonsynonymous substitutions between mosquito vitellogenin genes.

**Table 8.**
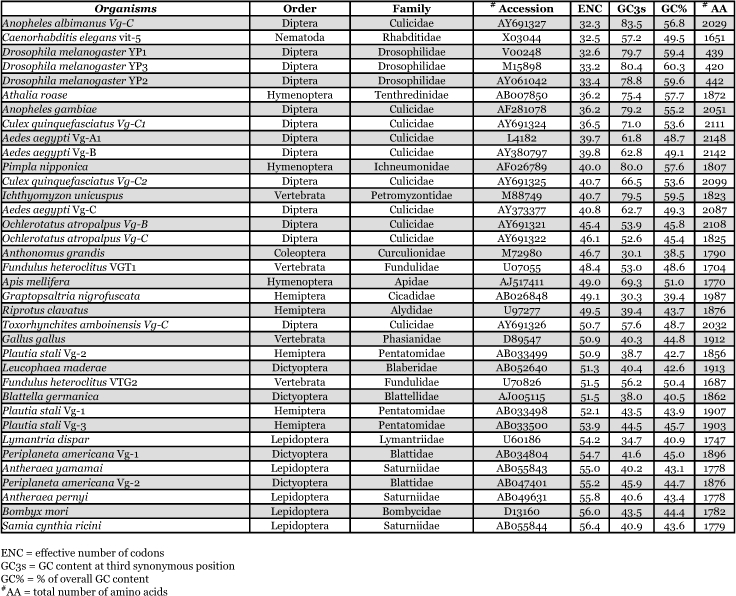
Effective number of synonymous codons of vitellogenin genes of insects and other organisms. Ordered by ENC value.

The pattern of synonymous codon usage is also of interest. Since the overall synonymous codon usage provides less informative data, Table 9 includes gene products with various expression levels. Preferred and rare codons were determined from the top 15% of all genes based on ENC and vitellogenin genes, assuming that these gene products are highly expressed. Amino acids such as asparagine, lysine, glutamine, phenylalanine, and tyrosine are preferentially encoded by one codon and not by other synonymous codons. Genes that use synonynous codons more equally (have high ENC) are probably under weak translational selective constraints, as those gene products may not be highly expressed. Synonymous codon usage in *An. gambiae* ribosomal genes (ENC ≤ 40.8) was also examined (Table 11). An analysis of interspecific synonymous codon choice in vitellogenins and ribosomal proteins revealed that when bias is present most of the preferential codons were conserved, except for glutamic acid; *An. gambiae* ribosomal genes preferentially use GAG over GAA, and *Ae. aegypti* vitellogenin genes preferentially use GAA over GAG. Extremely rarely used synonymous codons such as TTA (leucine), CTA (leucine), ATA (isoleucine), GGG (glycine) are also evolutionary conserved, suggesting that the usage of these codons may be disadvantageous to the translational rate of highly expressed proteins.

The patterns of synonymous codon usage in the autogenous and anautogenous mosquito vitellogenin genes were also examined (Table 12). All anautogenous mosquito vitellogenin genes (*An. albimanus*, *Ae. aegypti* and *Cx. quinquefaciatus*, shown in red in Table 12) revealed a high synonymous codon usage bias, preferentially using one or two synonymous codons over others. For example, amino acids such as asparagine, lysine, glutamine, phenylalanine, and tyrosine are almost exclusively encoded by one synonymous codon and not by other synonymous codons. An analysis of interspecific codon choice in anautogenous mosquito vitellogenin genes revealed that when bias is present most of the preferential codons were conserved in all three species. The exception is glutamic acid where *An. albimanus* Vg-C preferentially uses GAG over GAA, and all three vitellogenin genes in *Ae. aegypti* use GAA over GAG.

**Table 9.**
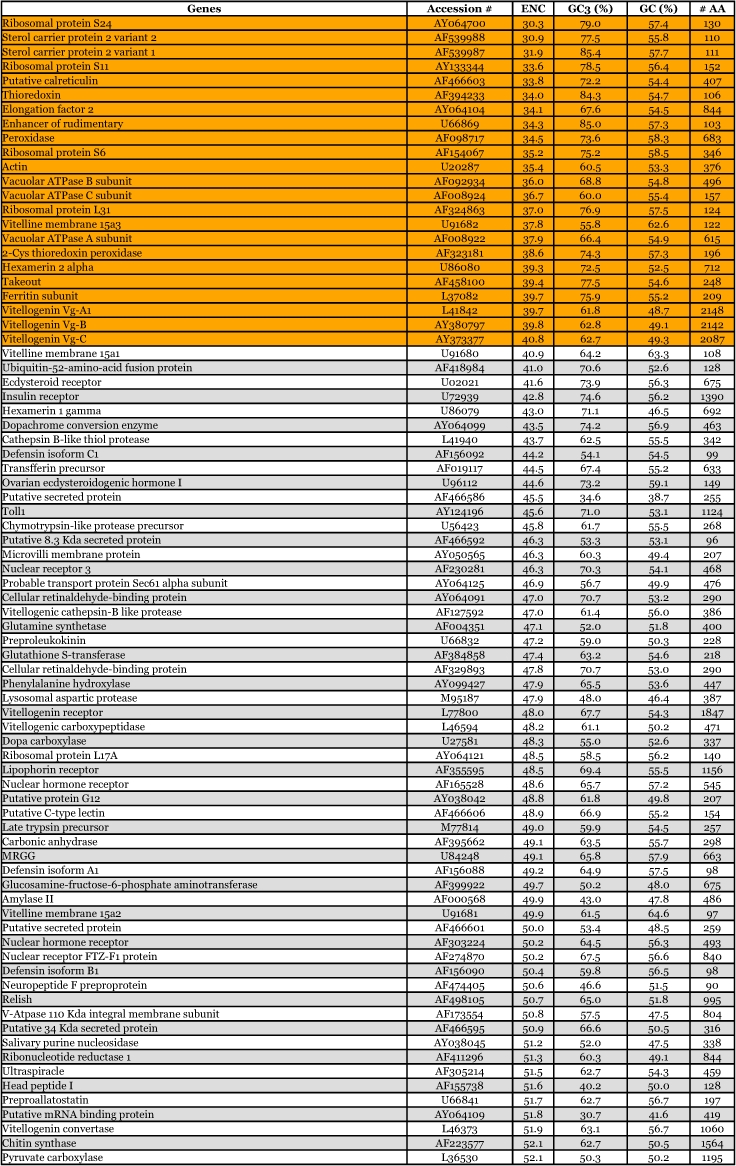
Synonymous codon usage bias of 150 *Aedes aegypti* genes, not including mitochondrial genes. Orange = top 15%. Blue = lower 15%.

**Continued:**
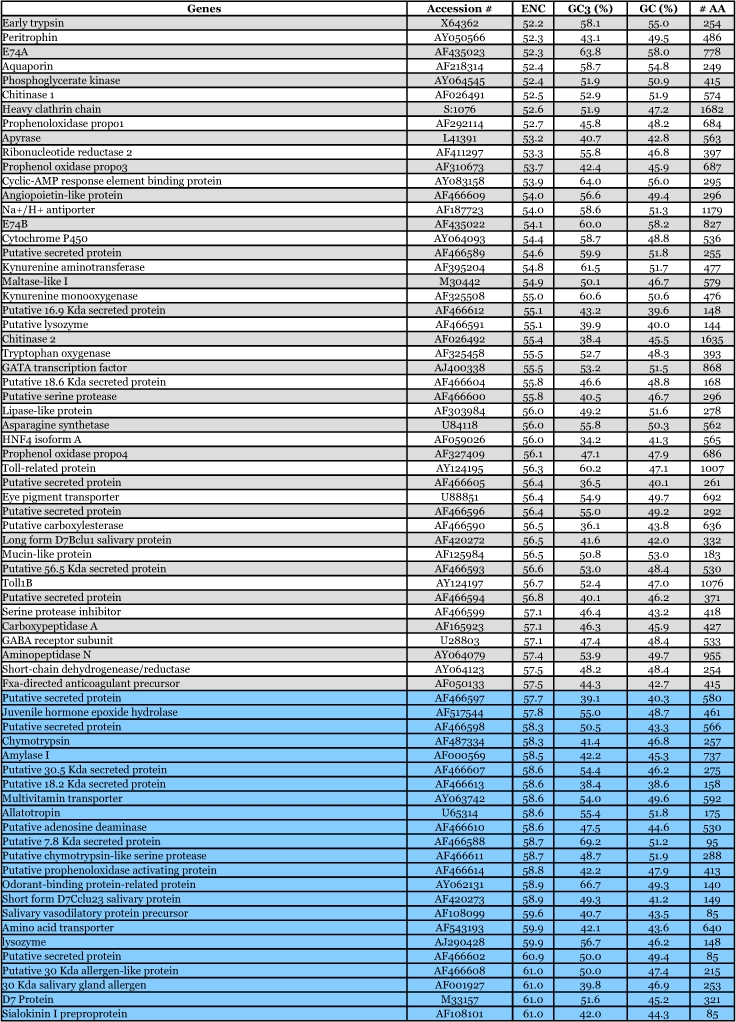

**Table 10.**
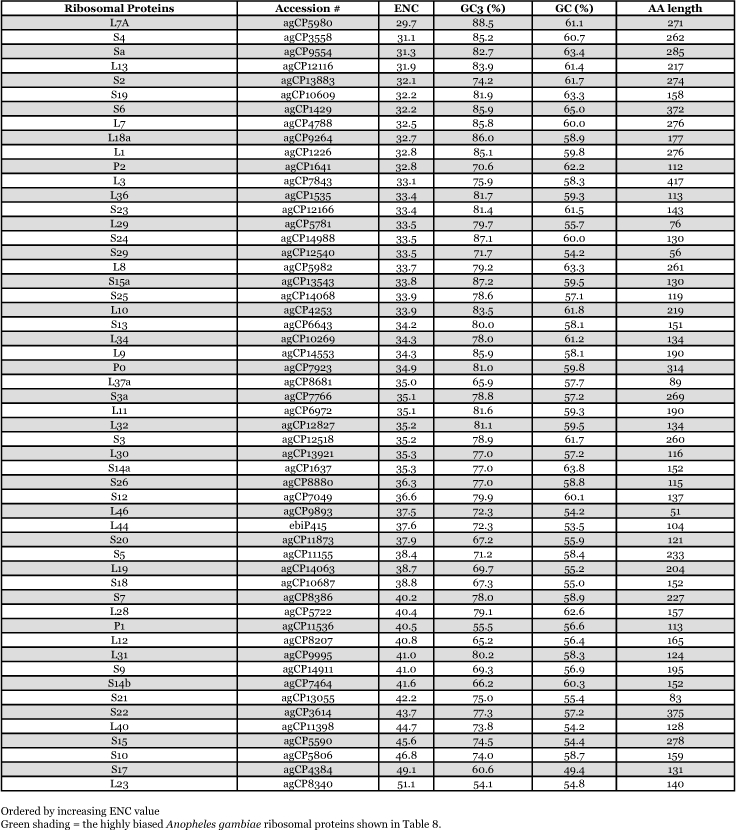
Synonymous codon usage bias for *Anopheles gambiae* ribosomal protein genes

In contrast, vitellogenin genes from autogenous mosquitoes (Oc. *atropalpus* and *Tx. amboinesis*, shown in blue in Table 12) showed a relatively low synonymous codon usage bias. *Tx. amboinensis* Vg-C, and Vg-B and Vg-C of *Oc. atropalpus*, showed the lowest bias among all mosquito vitellogenin genes examined. The average ENC for the two autogenous species was 47.4, and for the three anautogenous species was 38.6. A positive correlation was found between GC content and the degree of synonymous codon usage bias measured by ENC (Figure 9). Thus, the highest synonymous codon usage bias of *An*. *albimanus* Vg-C probably reflects the biased GC3s (83.5%). Complete sequences of 54 *Ae. aegypti* genes coding for other proteins were analyzed for synonymous codon usage bias. The vitellogenin genes show a pattern of codon usage that is among the most highly biased of all known complete *Ae. aegypti* genes based on ENC (Figure 9).

The nucleotide composition of the mosquito vitellogenin genes were also examined. A χ2 test of homogeneity of base frequency across taxa was performed using the PAUP* program to determine the level of bias at each codon position. This analysis showed that the base frequency for the first and third codon positions varied significantly across taxa (*p* < 0.01), probably due to different codon usage preferences among mosquito species.

**Figure 8.**
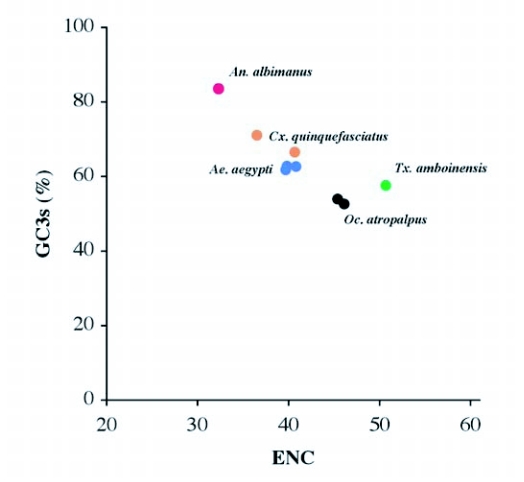
The correlation of synonymous codon usage bias and GC3 of anautogenous and autogenous mosquito vitellogenin genes.

### Vitellogenin gene expression in *Ae. aegypti*

Previous studies by Northern analysis and RNA dot blot analyses using a probe that most likely cross-hybridized with all members of the vitellogenin genes demonstrated that vitellogenin gene(s) were expressed only in fat body preparations of blood-fed female mosquitoes (Racciopi et al. 1986; Gemmill et al. 1986). To determine if all three genes were expressed, and to confirm the sex-and-stage specificity of their expression, real-time PCR was used. These experiments demonstrated that mRNAs from Vg-A1, Vg-B and Vg-C showed peak expression by 36 hours, and negligible by 72 hours after a blood meal (Fig. 11). Expression in whole body extracts of non-blood fed females, males, larvae and pupae of all three genes occurred but at 4 to 6 orders of magnitude lower levels; expression of Vg-A1 at 36 hours was 4 orders, Vg-B was 5 orders, and Vg-C was 6 orders of magnitude higher than in fat body of non-blood fed females.

**Table 11.**
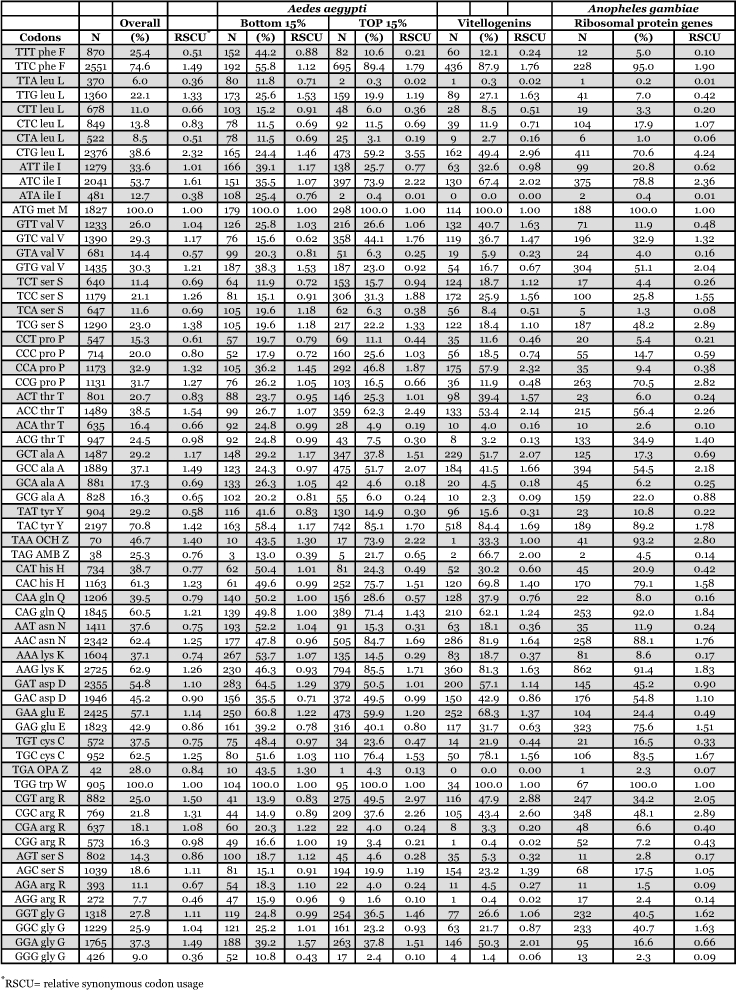
Synonymous codon usage of *Aedes aegypti* genes and *Anopheles gambiae* ribosomal protein genes, not including mitochondrial genes.

**Table 12.**
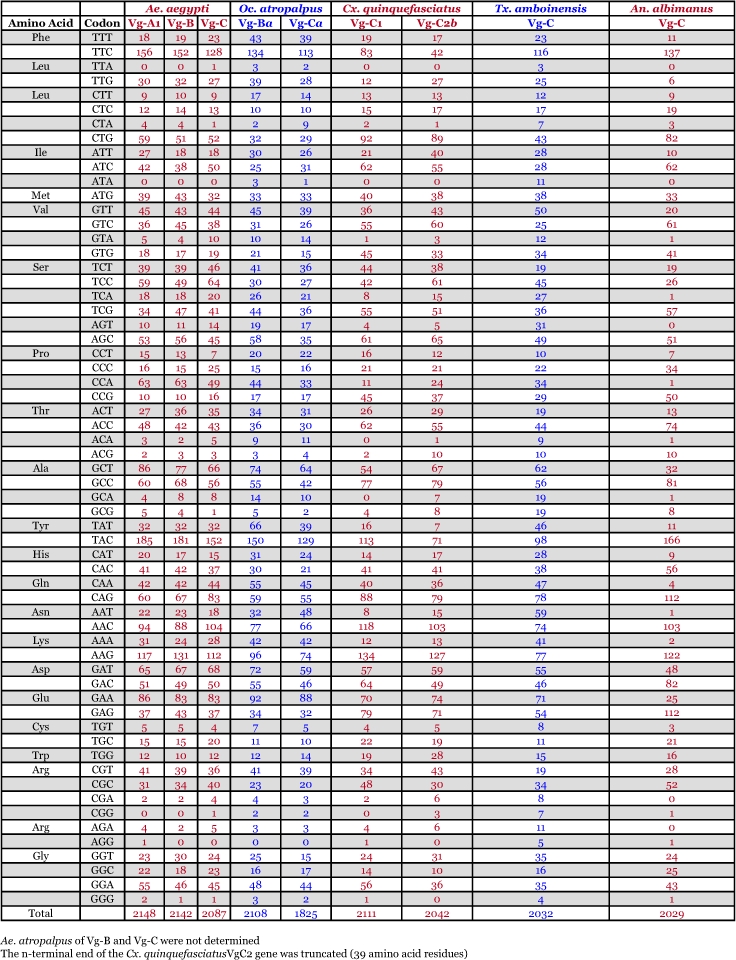
Synonymous codon usage in autogenous and anautogenous mosquito vitellogenin genes. Red = anautogenous. Blue = autogenous.

## Discussion

### Sequence analysis of vitellogenin genes in *Ae. aegypti*

To gain a better understanding of the evolution of vitellogenin genes in mosquitoes, genomic DNA libraries were constructed from several distantly related mosquitoes with different reproductive strategies, and vitellogenin genes were cloned and sequenced from them to conduct a comparative sequence analysis of mosquito vitellogenin genes.

**Figure 9.**
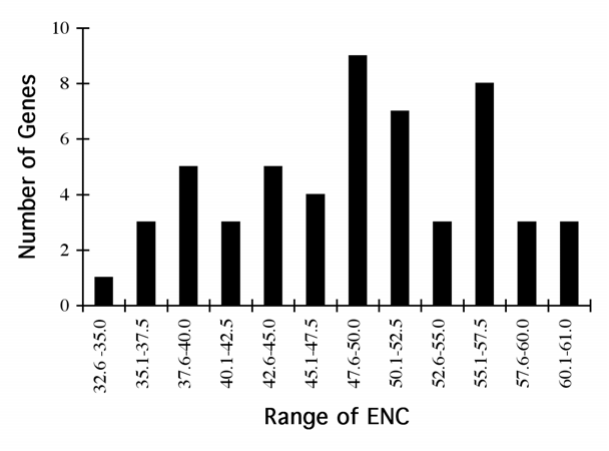
Distribution of *Aedes aegypti* genes based on synonymous codon usage bias. ENC = effective number of codons. Vg-A1, 39.7, Vg-B 39.8. Vg-C 40.8.

Four vitellogenin genes of *Ae. aegypti* had been previously cloned (Hamblin et al. 1987), and one of them, Vg-A1, was sequenced and extensively studied (Gemmill et al. 1986; Racioppi et al. 1986, Chen et al. 1994; Romans et al. 1995). However, the clones of the other genes were lost. To gain a better understanding of the evolution of these genes, genomic DNA libraries were screened. Four vitellogenin genes, Vg-A1, Vg-A2, Vg-B and Vg-C, were again cloned. Vg-A2, Vg-B and Vg-C were sequenced and a comparative sequence analysis was conducted. Sequence analyses of Vg-A1 and Vg-A2 showed that they are possibly allelic to each other, Vg-A1 and Vg-B are closely related, and possibly arose by a recent gene duplication event, and Vg-C is distantly related to Vg-A1 and Vg-B lineage, and possibly arose by an earlier gene duplication event.

**Figure 10.**
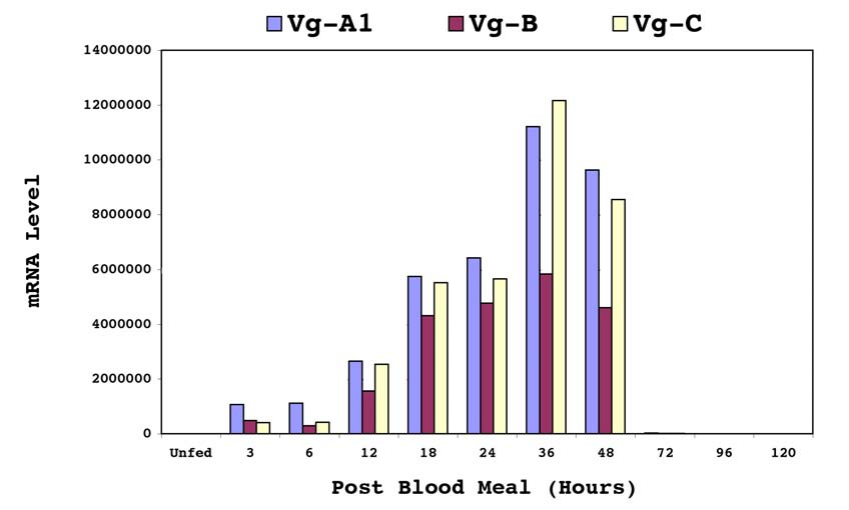
Expression of *Aedes aegypti* vitellogenin genes. mRNA levels were measured by real-time PCR.

Vg-B and Vg-C were relatively conserved in length with minor insertions and deletions of amino acids, especially within the polyserine regions, relative to Vg-A1. Molecular signatures previously characterized in Vg-A1 were all present and conserved. These include two short introns, two polyserine regions, highly conserved cysteine amino acid residues, and a cleavage site between the two vitellogenin subunits. The vitellogenins are postranslationally cleaved in the fat body into two small and large subunits (Romans et al. 1995) (Figure 1) by a vitellogenin convertase (Chen and Raikhel 1996). The predicted cleavage sequence (RXRR, Hagedorn et al 1998; Sappington and Raikhel 1998) was present in all three vitellogenins. The position of cysteine residues are also well conserved. There are 19 evolutionarily conserved cysteines out of the 20 residues in Vg-A1 and Vg-B, and out of 24 in Vg-C. In general, disulfide bonds between cysteine amino acid residues stabilize protein structure. When vitellogenin protein sequences from other insect taxa were compared to the *Ae. aegypti* sequences for the position of cysteine residues, 6 positions near the C-terminal were found to be highly conserved, suggesting that the cysteines at these positions may play a role in maintaining the structure of the vitellogenin proteins of insects.

### Sequence analysis of vitellogenin genes in other mosquitoes

Two distinct members of the vitellogenin gene family, Vg-C1 and Vg-C2, were cloned from *Cx. quinquefasciatus.* A complete coding sequence was obtained for Vg-C1, but the Vg-C2 sequence is truncated at the N-terminal end such that 39 amino acid residues are deleted. The nucleotide position at the truncation differs from the conserved first intron site of other mosquito genes, and the nucleotide sequence at the truncation also differs from the TAG or CAG that are always present in the 3′ intron sequence of all mosquito vitellogenins, suggesting that it is unlikely that the truncation is due to the acquisition of a new intron site. Other organisms such as *G. gallus* (Silva et al. 1989) and *C. elegans* (Spieth et al. 1985) have pseudogenes encoding non-functional vitellogenins due to stop codons and frame shifts in the coding sequences. However, in *Cx. quinquefasciatus* Vg-C2, the rest of the coding sequence has no stop codon or frame shift, suggesting that the gene could be still active. The appearance of the truncation could be due to the insertion of transposable elements that are relatively abundant in the genome of mosquitoes (Tu 1999). Sequencing the upstream region of the truncation of Vg-C2, and expression studies either by a reverse transcription coupled with polymerase chain reaction, or 5′ rapid amplification of cDNA ends using gene specific primers, would reveal whether *Cx. quinquefasciatus* Vg-C2 is expressed. If it is indeed expressed, it would be interesting to compare the pattern of expression with Vg-C1 that does not have a truncation.

Screening of An. *albimanus* and *Tx. amboinensis* genomic libraries resulted in positive clones that were all Vg-C orthologs based on the presence of molecular signatures. Rescreening with Vg-C probes derived from both species at a low stringency did not identify other members of the vitellogenin gene family. It is not certain whether the Vg-A1/B members became pseudogene(s), or the rate of mutation in this lineage is significantly different from others such that screening of the distantly related *Cx. quinquefasciatus*, *Tx*, *amboinensis*, and *An. albimanus* libraries failed. This is unlike other organisms such as *X. laevis* (Germond et al. 1984), *C. elegans* (Spieth et al. 1991), *D. melanogaster* (Wahli 1988), and *Ae. aegypti* (Hamblin et al. 1987), in which several members of the vitellogenin gene family exist.

However the Hymenoptera have a single vitellogenin gene (Kageyama 1994; Nose 1997; Piulachs et al. 2003; Donnell 2004).

All vitellogenin genes with complete coding sequences cloned in this study were relatively conserved in length with minor insertions and deletions of amino acids, especially within the polyserine regions, relative to their orthologs in *Ae. aegypti.* Molecular signatures previously characterized in *Ae. aegypti* were all present and conserved in the vitellogenin genes of these mosquitoes.

### Amino acid composition

The vitellogenin proteins provide amino acid building blocks for embryonic development and therefore the precise amino acid sequence could be of minor importance. However, the comparative sequence analysis of vitellogenin genes suggests that a majority of amino acid substitutions are due to conserved and moderately conserved changes in amino acid physical-chemical properties. As described above, the position of cysteine residues in all vitellogenin genes is highly conserved, consistent with other comparative studies including distantly diverged organisms (Wahli 1988; Byrne 1989, Trewitt et al. 1992; Hagedorn et al. 1998), suggesting that the overall tertiary structure of the vitellogenin proteins is important. If the amino acid sequence were of minor importance, there would likely be low discrimination between the extent of amino acid substitutions and their physical and chemical properties. Taken together, the analysis of *Ae. aegypti* vitellogenin proteins suggest that they are under moderate selective constraints to maintain tertiary structure in parts of the molecule, particularly by conserved cysteine residues. This conclusion is strongly supported by the anaysis of the tertiary structure of Lamprey vitellogenin by X-ray crystallography (Raag et al. 1988; Sharrock et al. 1992; Thompson and Banaszak 2002), which revealed a dimer that formed a pocket that could contain lipid. This region of the protein is highly conserved in the vitellogenins (Hagedorn et al. 1998).

Amino acid compositional analysis of the *Ae. aegypti* vitellogenin proteins revealed specific amino acid usage. All mosquito vitellogenin proteins, except for two vitellogenins cloned from *Cx. quinquefasciatus*, have a higher content of the aromatic amino acid residues tyrosine and phenylalanine than vitellogenins of other organisms. It is also interesting that the amino acid composition of all complete sequences available for non-vitellogenin proteins of *Ae. aegypti* shows that none of them exhibits the high content of aromatic residues seen in vitellogenins, except for the hexamerin storage proteins. The biological roles of storage proteins have been investigated, especially in several holometabolous insects. Two important physiological processes, metamorphosis and female reproduction, have been shown to be linked with the utilization of hexamerin storage proteins (Haunerland 1996). In holometabolous insects, storage proteins accumulate before the metamorphic molt of the last larval instar and are known to play an important role in adult cuticle formation by providing tyrosine residues as precursors for tanning.

A female-specific hexamerin storage protein has been identified from autogenous *Oc. atropalpus* mosquitoes, and this protein is believed to provide amino acid residues for vitellogenin protein synthesis by the fat body in the absence of protein from a blood meal (Wheeler and Buck 1996). Assuming that autogenous mosquitoes and other autogenous nematoceran insects, such as chaoborid midges, share common ancestors as is suggested by molecular data (Miller et al. 1997), these ancestors must have relied on storage proteins accumulated from the larval stages for vitellogenin synthesis. Once mosquitoes secured the anautogenous mode of reproduction, the proteinaceous blood meal was utilized for vitellogenin synthesis, and hexamerin storage proteins were no longer utilized. The evolutionarily and structurally unrelated storage proteins and vitellogenins may have a common biological role in providing precursors for embryonic cuticle formation in autogenous and anautogenous mosquitoes as shown in their high content of tyrosine and phenylalanine, although the vitellogenin genes of other insects are not high in these aromatic amino acids. However, why *Cx. quinquefasciatus* vitellogenins break the pattern of high aromatic amino acids in mosquitoes remains an unresolved question.

### Synonymous codon usage

The mosquito vitellogenin genes were shown to have high synonymous codon usage bias, preferentially using one or two “optimal” synonymous codons over others. This codon bias phenomenon is common to highly expressed genes in other organisms. As has been suggested in unicellular organisms such as *E. coli* and *S. cerevisiae*, species-specific optimal codons have been used selectively for efficient translation elongation in highly expressed genes since the abundance of each isoaccepting tRNA reflects the corresponding optimal codon in a species-specific manner (Ikemura 1981; Gouy and Gauthier 1982; Benzene and Hall 1982). Thus, the results obtained in this study are in a good agreement with results from other highly expressed genes in suggesting that the highly expressed vitellogenin genes also exhibit high synonymous codon usage bias (Kurland 1991; Ikemura 1985). The GC content at the third codon position has been regarded as one of the major factors influencing synonymous codon usage bias in *D. melanogaster* (Shields at al. 1988; Powell and Moriyama 1997).

The single vitellogenin gene (Vg-C) cloned from *An. albimanus* shows the highest codon bias among all mosquito vitellogenin genes with an ENC of 32.2. Among four other complete gene sequences available from *An. albimanus*, one of those, heat shock protein 82 gene (hsp82, accession number: L47285), also exhibits a similar degree of synonymous codon usage bias (ENC: 32.8) with very similar patterns of optimal codon usage. The GC content at the third codon position has been regarded as one of the major factors influencing synonymous codon usage bias in *D. melanogaster* (Shields at al. 1988; Powell and Moriyama 1997). This also appears to be the case with the mosquito vitellogenin genes. An extreme codon usage bias was found in the codons for asparagine, lysine, glutamine, phenylalanine, and tyrosine amino acid residues in Vg-C where over 90% of these amino acids were coded by a single optimal synonymous codon with G or C in the third position. Thus, the bias in highly expressed *An. albimanus* Vg-C and heat shock protein 82 probably resulted from the biased GC content at the third codon position, which is 83.5% and 81.3%, respectively. Although more complete sequences from highly expressed genes in *An. albimanus* are required to deduce optimal codons in this species because the abundance of each tRNA has not been investigated from mosquitoes, other Anopheline species such as *An. gambiae* also show very high GC content at the third codon position (Besansky 1993; Caccone et al. 1999). Vitellogenin genes of other anautogenous mosquitoes, *Ae. aegypti* and *Cx. quinquefasciatus*, also show high codon usage bias and high GC content at the third codon position.

However, vitellogenin genes of two autogenous mosquito species, *Oc. atropalpus* and *Tx. amboinensis*, show lower codon usage bias and GC content at the third codon position compared to those of anautogenous mosquitoes. The reason for the low synonymous codon usage bias of vitellogenin genes in autogenous mosquitoes could be that the amount of vitellogenin synthesized in these autogenous mosquitoes might be significantly smaller than anautogenous egg production, as shown in lower fecundity of facultatively autogenous mosquitoes (Magnarelli 1978; Jones 1993). Therefore, there may be no selective advantage to accumulate optimal codons for faster translation of vitellogenin mRNA. In the case of *Tx. amboinensis*, which live up to 80 days with continuous oviposition (Linley 1987; Tikasingh and Martinez 1991), the translational rate of vitellogenin could be much slower. Thus, the vitellogenin genes in these autogenous mosquitoes may be translated at a lower rate and therefore show lower synonymous codon usage bias.

When the intragenic position of rarely used synonymous codons in mosquito vitellogenin genes was examined, it was found that these codons are not randomly distributed. The conserved position of an extremely rarely used GGG synonymous codon coding for a glycine residue was found in the 12th position within the signal peptide region in all mosquito species examined, except in Vg-C of the autogenous mosquito *Tx. amboinensis* vitellogenin gene, where it was replaced by a preferential codon, GGA, coding for the same glycine residue. While there are about 100 glycine residues coded by the three other synonymous codons present throughout the protein, only one or two GGG codons were used, showing that the isoaccepting tRNA for GGG is very rare in mosquito vitellogenins. Subsequent analysis of intragenic positions of synonymous codons of each mosquito vitellogenin gene revealed that other rarely used synonymous codons such as AAA, ATA, TTA, and CTA have accumulated in the signal peptides of vitellogenin genes, suggesting that selective constraints act on this region to accumulate a cluster of rare synonymous codons in mosquito vitellogenin genes. Although it is not certain whether the presence of a cluster of rarely used synonymous codons in the signal peptide of mosquito vitellogenin genes is the result of selective functional constraints, or of site-specific differences in mutational rates, a potential selective mechanism that could explain the accumulation of rarely used synonymous codons at the N-terminal region of mosquito vitellogenin genes is presented below.

### Vitellogenin gene expression in *Ae. aegypti*

The fact that all three members of the vitellogenin gene family in *Ae. aegypti* are expressed in non-blood-fed females, males, larvae and pupae at trace levels, 4 to 6 orders of magnitude lower than in blood fed females, suggests that vitellogenin genes may be constitutively expressed in all stages prior to blood feeding. If there are not enough amino acids available to support the completion of oocyte maturation, it is energetically disadvantageous for female mosquitoes to utilize limited resources to synthesize an amount of vitellogenin proteins that would be insufficient for egg development. Thus, it is possible that a cluster of rare synonymous codons have selectively accumulated at the 5′ end of vitellogenin genes in anautogenous mosquitoes to regulate the rate of translation initiation that would down-regulate the level of vitellogenin protein synthesis in the absence of a blood meal. Once a blood meal is acquired and digested, there are enough amino acid residues available to be charged by tRNAs in lower abundance. In vertebrates, an estrogen-induced increase in specific tRNA for protein synthesis has been observed. For example, the level of specific serine isoaccepting tRNAs are differentially regulated in the liver of the chicken by estrogen during the synthesis of phosvitin, which contains a high number of polyserine residues (Maenpaa and Bernfield 1975). Thus, by extension, it is possible that ecdysone stimulates an increase in tRNA levels, particularly those required to increase translation of rare codons, in addition to its role in inducing transcription. Given the presence of polyserine regions in mosquitoes a similar up regulation of serine tRNA may also occur in response to ecdysone.

## Editor's Note

Dr. H. Fred Nijhout, Duke University, acted as editor for this paper.

## Figures and Tables

**Appendix 1:**
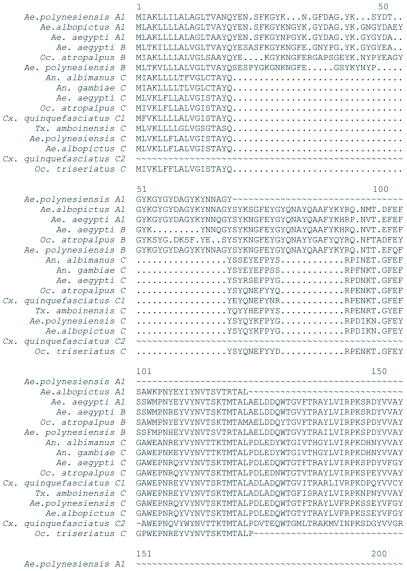
Alignment of the deduced amino acid sequences of mosquito vitellogenin genes. Conserved cysteine residues are highlighted. (…) indicate gaps inserted in the sequence. (∼∼∼∼) indicate missing sequences. Only partial sequences were obtained for *Ae. albopictus, Oc. triseriatus, and Ae. polynesiensis.*

## Abbreviations

GC3: G+C content 3rd position of the synonymous codon
ENC: effective number of codons
Vg: vitellogenin, Fat
body preparation: abdominal body wall with fat
